# Performance Evaluation Method for Intelligent Computing Components for Space Applications

**DOI:** 10.3390/s24010145

**Published:** 2023-12-27

**Authors:** Man Xie, Lianguo Wang, Miao Ma, Pengfei Zhang

**Affiliations:** 1 National Space Science Center, Chinese Academy of Sciences, Beijing 100190, China; xieman21@mails.ucas.ac.cn (M.X.); mamiao@nssc.ac.cn (M.M.); zhangpengfei22@mails.ucas.ac.cn (P.Z.); 2 University of Chinese Academy of Sciences, Beijing 100049, China

**Keywords:** embedded GPU, loongson platform, high-performance computing, performance evaluation

## Abstract

The computational performance requirements of space payloads are constantly increasing, and the redevelopment of space-grade processors requires a significant amount of time and is costly. This study investigates performance evaluation benchmarks for processors designed for various application scenarios. It also constructs benchmark modules and typical space application benchmarks specifically tailored for the space domain. Furthermore, the study systematically evaluates and analyzes the performance of NVIDIA Jetson AGX Xavier platform and Loongson platforms to identify processors that are suitable for space missions. The experimental results of the evaluation demonstrate that Jetson AGX Xavier performs exceptionally well and consumes less power during dense computations. The Loongson platform can achieve 80% of Xavier’s performance in certain parallel optimized computations, surpassing Xavier’s performance at the expense of higher power consumption.

## 1. Introduction

In recent years, with the increasing demand for space exploration, future space science missions will use larger sensors, higher sampling frequencies, and more accurate instruments compared to those in existing space science missions. Tasks such as autonomous exploration of robots and active debris removal require highly autonomous operations, which require more powerful on-board processing capabilities [1,2,3,4]. Traditional processors used for space missions cannot meet these high-performance requirements, and the process of redeveloping, producing, and certifying space-grade processors is lengthy, complex, and costly. Existing commercial off-the-shelf (COTS) devices have powerful parallel computing capabilities, and the application of artificial intelligence algorithms in space missions is becoming increasingly widespread. Commercial devices have high floating-point computational performance and excellent neural network accelerators, which can improve the efficiency and accuracy of space mission processing. Therefore, the aerospace field is currently searching for and evaluating processors that can meet high-performance requirements from existing commercial devices to alleviate these challenges. Some government aerospace agencies [5,6,7,8] and private enterprises [9,10] have conducted extensive research on performance evaluation of COTS devices. Some of these works focus on radiation studies of certain products, while others focus on balancing the performance of various architecture processors and determining which ones are suitable for space missions.

## 2. Related Work

Studies in references [11,12] evaluate the performance of COTS devices such as CPU, DSP, GPU, and FPGA based on data provided by suppliers. Using a set of established device metrics, different processor architectures are quantitatively analyzed and compared in terms of performance, power, efficiency, memory bandwidth, input–output bandwidth, and other aspects. However, vendor-supplied data and established metrics are often limited and lack consideration of deeper application directions such as space autonomous computing tasks. The Institute of Computing Technology of the Chinese Academy of Sciences, led by Luo Chunjie, has developed the mobile embedded device benchmark suite A IoT Bench [13], which aims to evaluate the artificial intelligence capabilities of mobile and embedded devices in image classification, speech recognition, converter translation, and micro-processing loads. The Embedded Microprocessor Benchmark Consortium (EEMBC) provides many benchmarks, such as ADAS Mark and ML Mark. The ADAS Mark benchmark is a performance measurement and optimization tool for building next-generation advanced driver assistance systems (ADAS) [14]. The image processing in this benchmark is close to what is carried out on spacecrafts, but it does not fully map to specific image processing tasks used in space applications. ML Mark is a benchmark for edge machine learning (ML) tasks [15]. ML workloads are trained based on space-independent ILSVRC2012 and COCO2014 datasets, and they do not consider typical sensor sizes used in space missions.

In the embedded CPU industry, the following three performance testing standards are widely recognized: Coremark, Dhrystone, and Coremark-Pro. Currently, Coremark-Pro has been used in the space domain, and examples of single-core and multi-core LEON processor (maintained by Gaisler Research) Coremark-Pro results are provided in [16,17]. Spacebench [18] encompasses a range of typical computations involving integers and floating-point numbers that are commonly employed in the realm of space computing. The open-source project GPU4S (GPU for Space), supported by the European Space Agency, provides a benchmark test that covers representative space algorithms in different space domains, used to evaluate GPU programming models for space payload processing [19]. OBPMARKs (On-Board Processing Benchmarks) was developed based on GPU4S and defines a set of benchmark testing methods covering common typical applications in space missions. Performance evaluations are carried out for high-performance processors such as ARM Mali G-72 (Cambridge, UK), NVIDIA Xavier NX (Santa Clara, CA, USA), and NVIDIA TX2 [20].

The computational performance requirements for space missions are constantly increasing, so a reasonable method for effectively characterizing the performance of new-generation COTS processors is essential. Based on the aforementioned investigation and analysis, the objective of this paper is to construct benchmarks for space applications and perform performance evaluations on currently available high-performance processors. The structure of the remaining part of this paper is as follows: Section 3 introduces the basic principles of performance evaluation, and determines benchmark testing methods and performance metrics; Section 4 presents the evaluation targets and experimental results of performance evaluation; finally, Section 5 provides a summary of the entire paper.

## 3. Evaluation Method

### 3.1. Overall Approach

**Figure 1:**
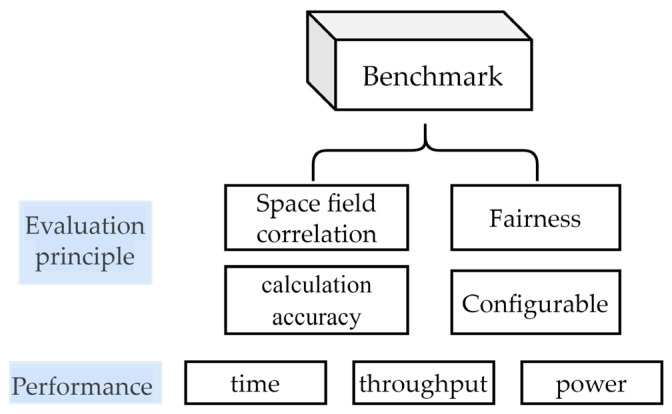
Basic principles of evaluation.

The purpose of this evaluation is to find a high-performance processor suitable for space applications. Therefore, the constructed benchmark tests need to be relevant to the space domain, using representative input data while covering as many space domains as possible. This ensures the correctness of the benchmark tests and that the implementation follows relevant standards, with corresponding reference outputs to check the correctness of the target platform’s output. This performance evaluation includes the CPU and GPU of the device, and it is conducted from multiple perspectives, as shown in Figure 1.

- The algorithms and input configurations in the benchmark need to cover existing spaceborne processing applications, as well as future scenarios and their performance requirements in different space domains;
- The benchmark tests should not be limited to a given processor, such as supporting a single programming model or architecture, but should be able to run on multiple platforms;
- The platform-independent parts of the benchmark should be the same for all platforms to enable fair comparisons;
- It is necessary to use common performance metrics for comparison. The performance metrics used in this evaluation include commonly used metrics such as total execution time, throughput, and FLOPS.

### 3.2. Common Benchmark—CoreMark-Pro Benchmark

First, this paper selects CoreMark-Pro benchmark tests for the pre-evaluation of processor performance. CoreMark-Pro benchmark tests include five popular integer workloads and four popular floating-point workloads. The integer workloads include JPEG compression, ZIP compression, XML parser, SHA-256 secure hash algorithm, and a memory-intensive version of the original CoreMark. The floating-point workloads include fast Fourier transform (FFT), linear algebra routines derived from LINPACK, a significantly improved version of the Livermore loop benchmark, and a neural network algorithm for pattern evaluation.

### 3.3. High-Performance Computing Benchmark

The complexity of current space missions is increasing, and high-performance computing is more and more widely applied to space missions, such as remote sensing data processing [21] and satellite visual navigation [22]. This work builds a diverse set of HPC-based benchmark tests; each benchmark is implemented in a parameterized way, and they are specified during the build configuration with CMake’s -D flag. The first parameter selects the type of benchmark to be compiled, and supports standard programming languages such as C, CUDA, and OpenMP; it then defines the data type, which, for all the benchmarks tested in this section, supports floating-point and double-precision floating-point numbers, and the last parameter defines the block size, which applies only to the GPU version of the code (this parameter is not required for OpenMP).

#### 3.3.1. Fast Fourier Transformation Benchmark (FFT)

Fast Fourier transform (FFT) is an algorithm that is more efficient in computing discrete Fourier transform (DFT). It is primarily used in communication and 2D image analysis. For example, in the Automatic Dependent Surveillance Broadcast (ADS-B) system, FFT is applied in a sliding window of 128 points to implement automatic monitoring technology for aircraft position determination through satellite navigation [23].

In this paper, the FFT benchmark first calculates a batch of predefined sizes of 1D FFT. The size parameter can be used to specify the size of these FFTs. A batch of FFTs is used to increase the overall execution time of the benchmark test, to reduce measurement errors, and to better utilize the kernel pipeline. The benchmark kernel is based on the cuFFT library provided by NVIDIA, with slight modifications to allow execution on the corresponding experimental platform, using complex single-precision floating-point values for computation. The configuration of the cuFFT library is carried out using FFTplan, where a plan defines a single transformation operation to be performed. With the plan, memory and computational resources can be pre-configured based on the size of the input data, allowing the processor to achieve optimal performance during actual computation.

#### 3.3.2. Finite Impulse Response Benchmark (FIR)

In the field of space signal processing, there is a growing demand for real-time and fast signal processing. Finite impulse response (FIR) filters are a type of filter structure that can be used to implement almost any type of frequency response digitally. FIR filters achieve filtering by using a series of delays, multipliers, and adders to create the filter’s output. The relationship between the output of an FIR filter with length N and the input time sequence x[n] is given by a finite convolution form, as shown below:

(1) $$\;y[n]=b_{0}x[n]+b_{1}x[n-1]+\cdots +b_{N}x[n-N]$$

(2) $$y[n]=\sum_{i=0}^{N}b_{i}x[n-i]$$

where x[n] is the input signal; y[n] is the output signal; $b_{i}$ represents the filter coefficients that constitute the impulse response, also known as tap weights.

N is the filter order; an *N*th order filter has N+1 terms on the right side. x[n−i] is commonly referred to as a tap, and based on the structure of tap delay lines, it provides delayed input to the multiplication operation in many implementation methods or block diagrams.

#### 3.3.3. Local Response Normalization Benchmark

Local response normalization (LRN) is a technique mainly used during deep learning training to improve accuracy, presented in 2012 by AlexNet [24]; its purpose is to perform local normalization on convolution values, and the specific calculation method can be found in Equation (3). LRN mimics the activity of biological neurons by creating a competitive mechanism, introducing competition between feature maps generated by adjacent convolution kernels. This makes significant features in feature maps more prominent in set A while being suppressed in adjacent feature maps, reducing the correlation between feature maps generated by different convolution kernels and enhancing the model’s generalization ability. The operation of LRN involves normalizing the pixel values at that point in the channel.

(3) $$b_{x,y}^{i}=a_{x,y}^{i}/{\left(k+\alpha \sum_{j=max\left(0,i-n/2\right)}^{min(N-1,i+n⁄2)}{\left(a_{x,y}^{j}\right)}^{2}\right)}^{\beta }$$

Using $a_{x,y}^{i}\;$ to denote the activity of a neuron computed by applying kernel i at position $\left(x,y\right)$ and then applying the ReLu (rectified linear unit) nonlinearity, N is the the total number of kernals. The constants k,n,α, and β are hyper-parameters, the values of which are determined using a validation set; we used $k=2,n=5,\alpha =10^{-4}$, and β=0.75.

#### 3.3.4. Matrix Multiplication Benchmark (GEMM)

Matrix multiplication is applied in many fields. In space exploration and astronomy, matrix multiplication is used to describe the motion of celestial bodies and analyze their orbits. Through matrix multiplication, the position, velocity, and orbital parameters of celestial bodies can be calculated for planetary orbit analysis, spacecraft navigation, and celestial dynamics research. The study in [25] documents the linear algebra benchmark performance of space processors using a 1024 × 1024 matrix.

The GEMM benchmark kernel is designed for matrix multiplication based on the Basic Linear Algebra Subroutines (BLAS) library and has been simplified. It is an optimized implementation of GPU matrix multiplication (GEMM) that is compatible with a wider range of devices. The GEMM benchmark tests the multiple applications of matrix multiplication; it calculates C=α·A·B+β·C, where $A,B,C\in R^{n\times n}$ and  α,β∈R. The floating-point operations of this benchmark are calculated as $\;2{\cdot n}^{3}$.

**Figure 2:**
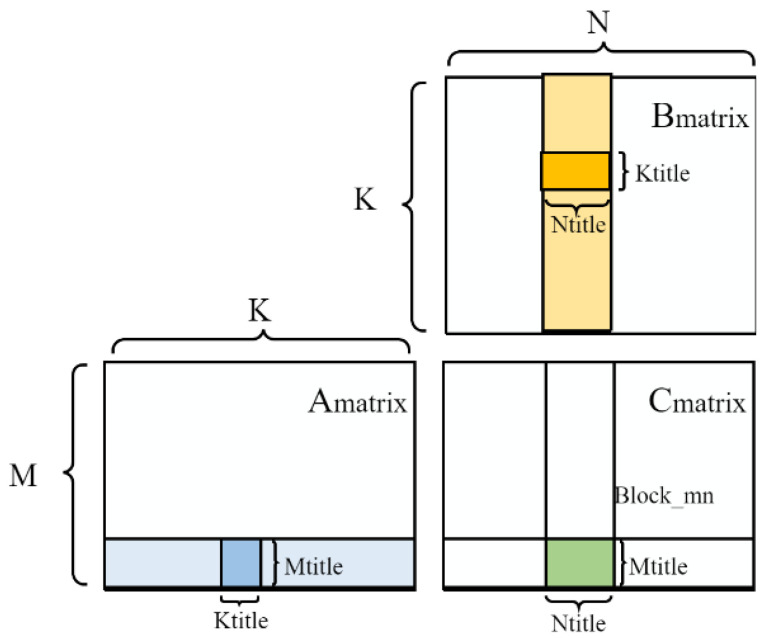
The layout method of the general matrix multiplication method.

The GPU implements GEMM by dividing the output matrix into tiles and then assigning small tiles to thread blocks. When calling cuBLAS with specific GEMM dimensions, the internal heuristic methods of cuBLAS can choose the tiling option expected to perform the best. The general tiling outer product method for matrix multiplication is shown in Figure 2.

#### 3.3.5. Convolution_2D and Correlation_2D Benchmark

Convolution_2D and Correlation_2D are used in combination with 2D images and form the foundation for implementing convolutional neural networks (CNNs), which can be used for visual-based navigation and image processing. In image processing tasks, the convolutional layer performs convolution operations on the input image by sliding a small matrix called a convolution kernel or filter, extracting specific feature information [26].

In this experiment, convolution and 2D correlation operations are implemented on the GPU using the CUDA Deep Neural Network (cuDNN) library provided by NVIDIA. cuDNN is a GPU acceleration library specifically designed for deep convolutional neural networks. By calling functions provided by cuDNN, such as cudnnConvolutionForward(), and cudnnPoolingForward(), and passing the corresponding handles, input data, weights, and output data descriptors, the corresponding convolution operations can be executed.

#### 3.3.6. Max Pooling and CIFAR-10 Benchmark

Max pooling is commonly used in various fields such as image processing, neural networks, and signal processing. It is also included in the soft-max and max-pool functions of the cuDNN library. The max pooling operation divides the entire image into non-overlapping blocks of the same size, and only retains the maximum value within each block, resulting in an output that maintains the original planar structure after discarding other nodes.

CIFAR-10 uses a 10-layer neural network for inference and is trained on the CIFAR-10 dataset [27]. Each layer is constructed by reusing neural network building blocks from various benchmarks. In the convolutional neural network model, data augmentation is applied to the training dataset, including random flipping, random cropping, and the normalization of input images, to generate more samples. L2 regularization is applied to the training weights, and a BN (batch normalization) layer is used after each convolutional layer to enhance the model’s generalization ability. Specifically, the BN operation transforms the activation values of each hidden layer neuron as follows:

(4) $${\hat{x}}^{\left(k\right)}=\frac{x^{\left(k\right)}-E[x^{\left(k\right)}]}{\sqrt{Var[x^{\left(k\right)}]}}$$

$x^{\left(k\right)}$ of a certain neuron in layer t does not refer to the original input, but rather to the linear activation function, of the neuron in layer t. During the training process, the distribution of input values in the internal layers of the network also changes continuously due to the change in parameters. Batch normalization (BN) normalizes the distribution of input values for any neuron in each layer of the neural network to a standard normal distribution with a mean of 0 and a variance of 1 through certain normalization methods.

### 3.4. Typical Application Benchmarks in Space

We define a set of benchmark testing methods that cover common typical applications in space missions. This article selects image calibration applications and AES encryption applications to construct the benchmark, reusing optimized parallel kernel implementation methods such as FFT and FIR filtering from the GPU4S benchmark.

#### 3.4.1. Image Calibration Application Benchmark

Image calibration has many important applications in the field of remote sensing and other space domains. Calibration can reduce noise, artifacts, and geometric distortions in images, and improve the color consistency and geometric accuracy of remote sensing images, thereby improving the quality and accuracy of remote sensing images and helping classification algorithms better accomplish their corresponding recognition and classification tasks. The thermal infrared band in remote sensing images can be used to estimate surface temperature. Image calibration can eliminate radiometric disturbances and non-linear sensor responses in images, thereby improving the accuracy of surface temperature estimation.

**Figure 3:**
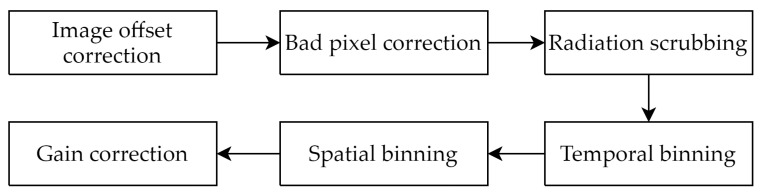
Image calibration processing flowchart.

In remote sensing applications with panchromatic sensors, image calibration is needed for images captured using imaging instruments on deep space exploration telescopes that require long exposures. Typically, to overcome the limitations of the sensor, multiple frames of images need to be acquired from the front end, and then they are overlaid and summed to form the final image. Before stacking the images, each frame of acquired images needs to undergo related preprocessing operations, namely image calibration testing. The specific steps are as shown Figure 3. When executing the benchmark experiments, three basic parameters of the image, “IMAGE_FRAMES, IMAGE_WIDTH and IMAGE_HEIGHT”, need to be entered.

#### 3.4.2. AES Encryption Application Benchmark

The AES (Advanced Encryption Standard) encryption algorithm has many applications in the field of aerospace and satellite, and is usually used to protect the security of communication data, storage data, and key exchange. For example, spacecraft and satellites will regularly send remote test data, such as posture information, temperature, and battery status. Using the AES encryption algorithm can ensure the security of these data during the transmission process, prevent data from being eavesdropped or tampered with, at the same time protecting the communication between the ground station and the spacecraft or satellites, and prevent unauthorized access and attacks. In addition, some spacecraft and storage devices on satellites include sensitive information, such as navigation data, images, and videos, using the AES encryption algorithm to ensure that these data are protected during the storage process.

**Figure 4:**
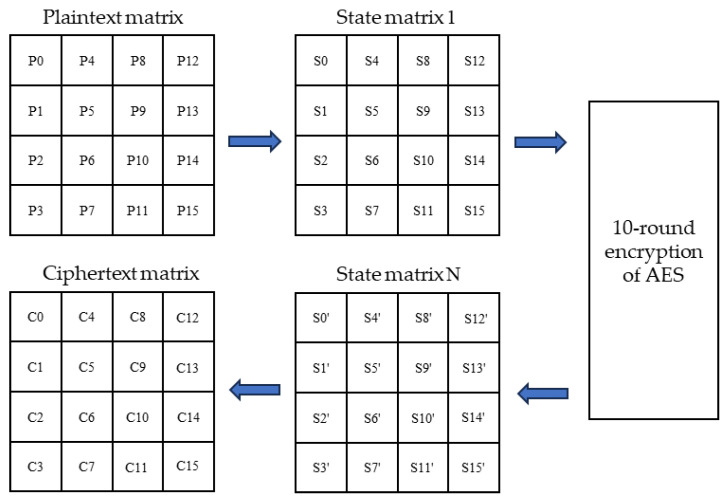
AES encryption algorithm execution process.

The standard AES algorithm is a symmetric-key encryption algorithm that encrypts the grouped plaintext by executing the same polling function 10 times, ultimately generating a ciphertext, which requires the same key for encrypting and decrypting the plaintext. The unit of processing in AES is the byte, and the input 128-bit plaintext (P) and the key (K) are divided into 16 bytes. In general, the grouped plaintext is described by a square matrix in bytes, called the state matrix. In each round of the algorithm, the content of the state matrix keeps changing and the final result is output as the ciphertext. The bytes in this matrix are arranged in order from top to bottom and left to right. The flow is shown in Figure 4 below. When executing the benchmark experiment, we need to input two basic parameters, “DATA_LENGTH and KEY_LENGTH”, which are the length of data to be encrypted and the length of the key.

## 4. Benchmark Execution and Evaluation

### 4.1. Experimental Hardware Platforms

Internationally, the commercial high-performance processor market is mainly dominated by three companies: NVIDIA, AMD (Santa Clara, CA, USA), and Intel (Santa Clara, CA, USA). In terms of processors for high-performance computing, AMD’s latest Instinct MI250X processor has a double-precision floating-point operation capability of up to 95.7 TFlops (trillions of floating-point operations per second), and NVIDIA’s latest Jetson Xavier series high-performance processors also achieve peak performance in the tens of the TFlops range [28], while the 4th Gen Intel Xeon Scalable processors will deliver improved performance, efficiency, and cost savings for targeted workloads of built-in accelerators. Among these three companies, NVIDIA has a more complete software and hardware ecosystem, providing the CUDA (Compute Unified Device Architecture) platform for developers to create parallel computing programs. In addition, NVIDIA provides various acceleration libraries for intelligent computing. High-performance processors mainly include Loongson series processors, Shenwei series processors, and Veiglo series processors. The Loongson processor is widely used in computer, server, and embedded systems, and has shown good performance and stability in certain specific application scenarios.

This article selects NVIDIA Jetson AGX Xavier, the Loongson platform (Beijing, China), and ASUS laptop processors (Taipei, Taiwan) for system evaluation and analysis. Among them, the ASUS laptop is mainly used as a reference for basic performance results. NVIDIA Jetson AGX Xavier is a heterogeneous SoC (system-on-chip), including CPU, GPU, and several other accelerators; the Loongson experimental platform consists of the Loongson 3A4000 CPU and the Veiglo BI-V100 GPU. Based on the benchmark testing methods constructed in Section 3, this performance evaluation experiment is carried out. The basic performance parameters of the experimental platform are shown in Table 1.

From the table above, we can see the performance parameters of the three experimental platforms, including CPU, GPU architecture, GPU memory, power consumption modes, etc. Comparing the performance parameters of each platform from the table, we can see that the GPU architecture of Jetson AGX Xavier is more advanced, supporting Tensor Cores, which can better meet the needs of large-scale computing. In addition, Jetson AGX Xavier has an optional 10W power consumption mode, consuming less power compared to the other two platforms. The Loongson platform has the highest number of CUDA cores and 32 GB HBM2 GPU memory, but its power consumption is several times that of other platforms.

### 4.2. CoreMark-Pro Benchmark Experiment Results

The CoreMark-Pro benchmark test measures the computational performance of a system by performing a series of basic arithmetic and logical operations. This benchmark can highlight the strengths and weaknesses of the processor. In this experiment, the configurations on the three platforms all use four multi-core CPUs for calculations, and the final CoreMark-PRO score is generated by processing them through Perl scripts. The ratio of single-core and multi-core experimental results is then calculated. The test results are shown in Table 2, where the scores represent the number of algorithm executions performed by the system in a fixed time.

A higher score means that the processor can perform more computing tasks in a given time, reflecting its stronger performance. From the table above, it can be seen that for multi-core experimental results, the ASUS FL8000 platform has the highest CPU score, followed by the Jetson AGX Xavier platform. The multi-core experimental scores of the Loongson platform are about half and one-third of that of the Jetson AGX Xavier platform and the ASUS FL8000 platform.

### 4.3. High-Performance Computing Benchmark Experiment Results

Based on the benchmark methods described in Section 3.3, experiments are carried out on each of the three platforms, where GPU computing is implemented in the CUDA framework and CPU computing is implemented in the OpenMP framework, meanwhile, with the following parameters being set: “DATATYPE = FLOAT” and “BLOCKSIZE = 32”. It also includes their respective optimization implementations. In the CUDA framework, CUDA’s data parallelism and model parallelism techniques are used to accelerate the training process, while relevant acceleration libraries (cuDNN, cuBLAS, etc.) are called for optimization. In the OpenMP framework, critical computational loops are marked as parallel regions using compile directives to achieve thread-level parallel computation, and data locality optimization is used to improve computation speed.

#### 4.3.1. Test Results on GPUs

The CUDA framework has advantages such as parallel computing capability, high-bandwidth memory, flexible kernel function programming, and optimized GPU acceleration libraries, making it capable of providing more efficient and faster computing capabilities for space artificial intelligence applications. The results in Table 3 are the average statistics after 20 experiments. The table displays the total execution time of 12 computational benchmark modules on the experimental platforms.

The GPUs used in the three experimental platforms are NVIDIA Volta GV10B, NVIDIA 940MX, and Veiglo BI-V100. From the table, it can be seen that for some simple computational modules, the execution time difference among the three platforms is relatively small. However, for complex computational modules like the CIFAR-10 inference chain, the computational power of Jetson AGX Xavier is fully utilized, and the execution time is 4 times faster than that of the ASUS FL8000 laptop and 10 times faster than that of the Loongson platform.

**Figure 5:**
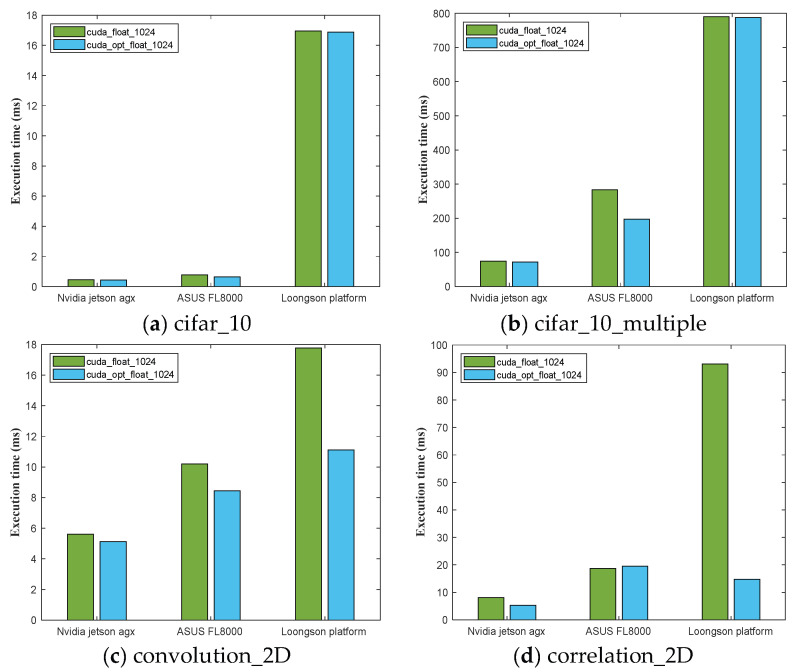
A comparison of the total execution time of the computational benchmark module based on the CUDA framework on the three experimental platforms, also including the optimization implementation, where (**a**) is the cifar_10 benchmark, (**b**) is the cifar_10_multiple benchmark, (**c**) is the convolution_2D benchmark, and (**d**) is the correlation_2D benchmark.

Figure 5 shows that Jetson AGX Xavier always has the fastest execution time, demonstrating its powerful computing performance. The Loongson experimental platform performed poorly in the inference of cifar_10. However, after optimized processing, the execution time of convolution_2D and correlation_2D operations was significantly shortened, and the difference with Jetson AGX Xavier was significantly reduced. This indicates that the Loongson platform also has certain advantages in some specific operations.

#### 4.3.2. Test Results on CPUs

OpenMP (open multi-processing) is an open parallel computing framework that can be used for multi-core CPU systems with shared memory architecture. It achieves the parallelization of tasks by using compiler directives and provides a set of API interfaces for writing parallel programs in a multi-threaded environment. The OpenMP framework has advantages such as simplicity, cross-platform compatibility, automated parallelization, and performance scalability. It can provide convenience and efficiency in CPU development for space applications, accelerate the computation process, and improve application performance.

Optimized implementations of OpenMP were also performed, and the average results of 20 experiments are shown in Table 4. The table shows the total program execution time of 12 computing benchmark modules on the experimental platforms. The CPUs of the three experimental platforms are 8-core Nvidia Carmel, 4-core Intel i7-8550U, and Loongson 3A4000. This experiment used four multi-core CPUs for computation on all three platforms.

From the experimental results in Table 4, it can be observed that the performance of the Nvidia Carmel CPU is very close to that of the Intel i7-8550U CPU, and overall, the performance of the Intel i7-8550U CPU is slightly stronger.

**Figure 6:**
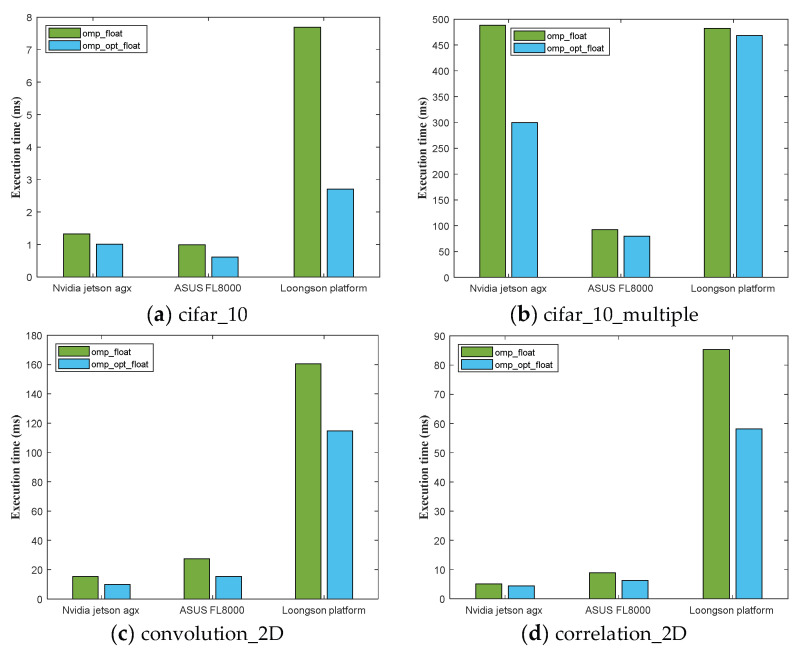
A comparison of the total execution time of the computational benchmark modules based on the OpenMP framework on the three experimental platforms, also including the optimization implementation, where (**a**) is the cifar_10 benchmark, (**b**) is the cifar_10_multiple benchmark, (**c**) is the convolution_2D benchmark, and (**d**) is the correlation_2D benchmark.

To present the above results more intuitively, the experimental results of the OpenMP framework were plotted into a bar chart using MATLAB (2018.a) software, as shown in Figure 6. From the graph, it can be seen that the execution time of the Loongson platform is relatively longer, at about five times longer than that of the other two platforms, and there is still a certain gap even after optimization. The main reason for this difference may be that the CPU of the Loongson platform is designed for desktop and mobile computing, focusing on comprehensive performance and general computing capabilities. On the other hand, Jetson AGX Xavier is designed specifically for embedded devices and edge computing, emphasizing low power consumption and high efficiency.

### 4.4. Typical Application Benchmark Experimental Results

Based on the two typical benchmarks introduced in Section 3.4 of this paper, experiments were conducted on three platforms, including implementations based on the CUDA framework on GPU processors, the OpenMP framework on CPU processors, and statistics on the throughput and power consumption of each platform during benchmark testing.

#### 4.4.1. Image Calibration Benchmark Test Results

The throughput and power results of the image calibration experiment are shown in Table 5 and Table 6. To improve the accuracy of the experiments and avoid exceptional cases, an average of 20 experimental results was taken for each platform under the same experimental conditions, measured in Mpixels/s. Five standard image sizes were used in the experiment: 1024 × 1024, 2048 × 2048, 4096 × 4096, 8192 × 8192, and 10240 × 10240. Fixed seed-generated quasi-random data were used as input data.

**Figure 7:**
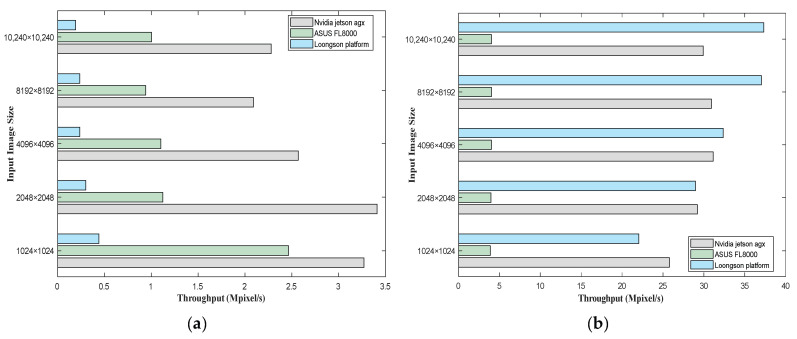
A comparison of the throughput computation results in Mpixels/s for the image calibration benchmarks executed on the three experimental platforms, where (**a**) is the OpenMP framework and (**b**) is the CUDA framework, computed by averaging the entire processing pipeline over 20 iterations.

In Table 5, the throughput of the CUDA framework is greater than that of the OpenMP framework for each experimental platform when inputting an image of the same size, and the difference in the execution results is more significant for the Loongson platform. Under the OpenMP framework, the throughput of the Jetson AGX Xavier platform is always the largest, but under the CUDA framework, the throughput of the Loongson platform surpasses that of Jetson AGX Xavier when the input image size increases to 4096 × 4096, though the power consumption of the Loongson platform is higher as well. In Table 6, when the input image size is 10,240 × 10,240, the power consumption of the Loongson platform is 4.67 times that of Jetson AGX Xavier (in the CUDA framework). Figure 7 presents a comparison that is more user-friendly and easier to understand.

#### 4.4.2. AES Encryption Algorithm Benchmark Test Results

The AES encryption algorithm experiment with encrypting data of different lengths using three key lengths: 128-bit, 192-bit, and 256-bit. To improve the accuracy of the experiments and avoid exceptional cases, an average of 20 experimental results was taken for each platform under the same experimental conditions. The throughput and power results of the AES encryption algorithm experiment on three platforms are shown in Table 7 and Table 8 below.

**Figure 8:**
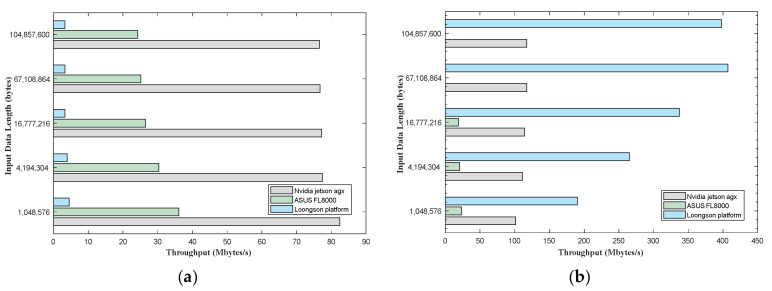
A comparison of throughput computation results in Mbytes/s for the AES encryption algorithm benchmark executed on three experimental platforms, where (**a**) is the OpenMP framework and (**b**) is the CUDA framework, which is computed by averaging the entire processing pipeline over 20 iterations.

According to the results shown in Table 7 and Table 8, during the benchmark testing of the AES encryption algorithm, it can be observed that for the OpenMP framework, the throughput of the Jetson AGX Xavier platform is 23 times higher than that of the Loongson platform when the encryption data size is 16,777,216 bytes. On the other hand, for the CUDA framework, the Loongson platform exhibits the highest throughput, with a throughput that is 3.5 times higher than that of the AGX Xavier experimental platform when the encryption data size is 67,108,864 bytes; however, the power is also 6.7 times that of the Jetson. A more visual comparison is illustrated in Figure 8.

### 4.5. Data Transmission Time Analysis

**Figure 9:**
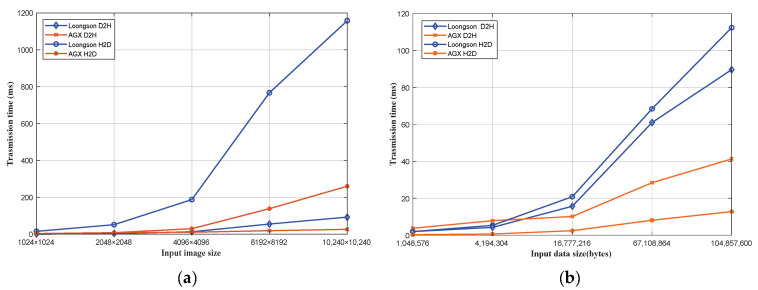
A comparison of the data transfer time (device to host; host to device) for the benchmarks on the experimental platform. where (**a**) is the Image calibration benchmark and (**b**) is the AES encryption algorithm benchmark.

In this paper, the data transmission time of the image calibration experiment and AES encryption experiment results of the Jetson AGX Xavier and Loongson platforms were analyzed. The GPU of the experimental platform is NVIDIA Volta GV10B and Veiglo BI-V100. Figure 9a shows the data transmission time comparison of the image calibration experiment, and Figure 9b shows the data transmission time comparison of the AES encryption experiment. D2H (device to host) represents the transfer of data from the device side to the host side, and H2D (host to device) represents the transfer of data from the host side to the device side. When the size of the transferred data is small, the data transmission time of the two platforms is similar. However, as the transferred data increase, the data transmission time of the Jetson AGX Xavier platform is much shorter than that of the Loongson platform. NVIDIA provides the interface of GPUDirect RDMA for professional-grade GPUs [29], which allows direct access to the bus address of GPU memory. When performing large-scale data transmission and computation, the Jetson AGX Xavier platform has advantages over the Loongson platform.

## 5. Conclusions

This study investigates benchmark tests for different application scenarios in ground and space missions. The benchmark tests for high-performance processors in space applications provided by the European Space Agency (ESA), GPU4S, and OBPMARK, were selected. The performance of the Jetson AGX Xavier embedded processor, Loongson platform, and Asus FL8000 laptop was systematically tested. We conducted tests on representative computational modules and application scenarios on the CPU and GPU of the three experimental platforms, including fast Fourier transform, matrix multiplication, CIFAR-10 inference chain, image calibration processing, AES encryption, etc. The performance of the Jetson AGX Xavier and Loongson platforms on different computationally intensive operators was analyzed in detail.

Through experimental research on the implementation ability of these architectures and the advantages of different architectures in space missions, this study provides important references for gradually adopting more advanced architectures to better meet the computational requirements of future space missions.

## Figures and Tables

**Table 1:** Basic performance parameters of experimental platforms.

Parameter	Jetson AGX Xavier	ASUS FL8000	Loongson Platform
CPU	Nvidia Carmel 8-core 2.3 GHz	i7-8550U 4-core 1.8 GHz	3A4000 4-core 1.8 GHz
Three-level cache	4 MB	8 MB	8 MB
GPU	Volta GV10B 1.4 GHz	Maxwell 940MX 1.2 GHz	Veiglo BI-V100 1.5 GHz
CUDA cores	512 CUDA cores	384 CUDA cores	3072 CUDA cores
Tensor cores	Support	Not Support	Not Support
GPU memory	32 GB DDR4 Sharing	2 GB GDDR5 Independent	32 GB HBM2 Independent
Power	30 W/15 W/10 W	15–200 W	220 W
CUDA version	CUDA11.4	CUDA11.4	CUDA10.2

**Table 2:** CoreMark-Pro experiment results (unit: iter/s).

Calculate Platform	Multi-Core	Single-Core	Scaling
Jetson AGX Xavier	14,360.88	3734.97	3.84
ASUS FL8000	22,599.79	6525.45	3.46
Loongson platform	7040.61	1927	3.65

**Table 3:** Benchmark experiment results of CUDA framework (unit: ms).

Benchmark Name	Jetson AGX Xavier		ASUS FL8000		Loongson Platform	
	GPU (Cuda)	GPU (Cuda_Opt)	GPU (Cuda)	GPU (Cuda_Opt)	GPU (Cuda)	GPU (Cuda_Opt)
fast_fourier_ transform	4.705	0.400	2.984	0.294	30.962	3.601
finite_impulse_ response_filter	1.043	0.523	0.214	0.788	4.582	2.454
LRN_bench	5.361	4.269	7.149	8.907	14.206	10.532
matrix_multiplica- tion_bench	138.326	47.547	336.830	127.513	51.823	56.630
convolution_2D	5.606	5.119	10.203	8.450	17.766	11.121
correlation_2D	8.141	5.234	18.737	19.482	93.056	14.723
max_pooling	2.509	2.152	4.717	4.200	8.537	8.724
cifar_10	0.457	0.431	0.772	0.651	16.956	16.876
cifar_10_muiple	73.995	72.100	283.187	197.027	790.402	787.754
relu_bench	4.704	4.302	7.280	6.424	14.973	12.910
softmax_bench	6.827	4.305	10.679	7.025	44.284	12.680
wavelet_transform	0.221	0.287	0.293	0.431	5.713	1.672

**Table 4:** Benchmark experiment results of OpenMP framework (unit: ms).

Benchmark Name	Jetson AGX Xavier		ASUS FL8000		Loongson Platform	
	CPU (Omp)	CPU (Omp_Opt)	CPU (Omp)	CPU (Omp_opt)	CPU (Omp)	CPU (Omp_Opt)
fast_fourier_ transform	0.247	0.813	0.056	0.357	0.210	1.847
finite_impulse_ response_filter	1.295	0.675	0.860	0.595	2.936	1.714
LRN_bench	22.352	17.231	16.544	12.149	86.929	77.217
matrix_multiplica- tion_bench	1824.736	212.291	1592.956	236.281	2020.820	928.384
convolution_2D	15.341	9.861	27.403	15.344	160.522	114.807
correlation_2D	5.103	4.332	8.894	6.218	85.302	58.161
max_pooling	1.671	1.542	3.910	1.873	18.803	9.848
cifar_10	1.330	1.013	0.996	0.617	7.686	2.702
cifar_10_muiple	488.251	299.511	92.495	79.795	481.898	468.590
relu_bench	3.076	2.824	4.104	2.895	14.400	8.733
softmax_bench	5.662	4.890	12.829	7.033	54.569	53.433
wavelet_transform	1.255	0.689	0.891	0.353	6.032	1.680

**Table 5:** Throughput of each experimental platform under Image Calibration Benchmark (unit: Mpixels/s).

Image Size	Throughput					
	Jetson AGX Xavier		ASUS FL8000		Loongson Platform	
	CPU (Omp)	GPU (Cuda)	CPU (Omp)	GPU (Cuda)	CPU (Omp)	GPU (Cuda)
1024 × 1024	3.27	25.78	2.46	3.88	0.44	22.05
2048 × 2048	3.41	29.20	1.12	3.96	0.30	28.97
4096 × 4096	2.57	31.15	1.10	4.01	0.24	32.36
8192 × 8192	2.09	30.93	0.94	4.02	0.24	37.02
10,240 × 10,240	2.28	29.91	1.00	4.01	0.19	37.34

**Table 6:** Power Consumption of each experimental platform under Image Calibration Benchmark (unit: W).

Image Size	Power Consumption					
	Jetson AGX Xavier		ASUS FL8000		Loongson Platform	
	CPU (Omp)	GPU (Cuda)	CPU (Omp)	GPU (Cuda)	CPU (Omp)	GPU (Cuda)
1024 × 1024	10.85	8.7	11.2	21.5	82.3	80.9
2048 × 2048	17.1	9.9	20.6	26.8	84.6	83.55
4096 × 4096	20.5	14.7	25.9	29.3	85.3	90.5
8192 × 8192	21	20.1	27.2	32.6	86.6	93.6
10,240 × 10,240	22.6	21.6	29.3	37.9	89.1	100.9

**Table 7:** Throughput of each experimental platform under AES Encryption Algorithm Benchmark (unit: Mbytes/s).

Data Length (Bytes)	Throughput					
	Jetson AGX Xavier		ASUS FL8000		Loongson Platform	
	CPU (Omp)	GPU (Cuda)	CPU (Omp)	GPU (Cuda)	CPU (Omp)	GPU (Cuda)
1,048,576	82.44	101.42	36.09	23.40	4.51	190.23
4,194,304	77.43	111.37	30.36	20.76	3.91	265.05
16,777,216	77.21	114.32	26.50	18.79	3.30	337.07
67,108,864	76.74	117.38	25.16		3.32	407.02
104,857,600	76.55	117.34	24.28		3.30	397.66

**Table 8:** Power Consumption of each experimental platform under AES Encryption Algorithm Benchmark (unit: W).

Data Length (Bytes)	Power Consumption					
	Jetson AGX Xavier		ASUS FL8000		Loongson Platform	
	CPU (Omp)	GPU (Cuda)	CPU (Omp)	GPU (Cuda)	CPU (Omp)	GPU (Cuda)
1,048,576	5.15	5.2	9.8	16.9	77.6	79.5
4,194,304	6.65	6.8	28.7	20.7	82.9	81.2
16,777,216	8.8	9	31.2	27.2	84.3	84.6
67,108,864	14.5	14.9	36.8		84	99.1
104,857,600	15.6	17.94	39.9		84.7	106.8

Note: For input data of 67,108,864 bytes and 104,857,600 bytes, it exceeded the maximum computational capacity of the ASUS FL8000 notebook CUDA framework, so no statistical results were obtained.

## Data Availability

Data are contained within the article.
